# Programming and simulating chemical reaction networks on a surface

**DOI:** 10.1098/rsif.2019.0790

**Published:** 2020-05-27

**Authors:** Samuel Clamons, Lulu Qian, Erik Winfree

**Affiliations:** 1 Bioengineering, California Institute of Technology, Pasadena, CA 91125, USA; 2 Computer Science, California Institute of Technology, Pasadena, CA 91125, USA; 3 Computation and Neural Systems, California Institute of Technology, Pasadena, CA 91125, USA

**Keywords:** molecular programming, surface chemistry, nanotechnology

## Abstract

Models of well-mixed chemical reaction networks (CRNs) have provided a solid foundation for the study of programmable molecular systems, but the importance of spatial organization in such systems has increasingly been recognized. In this paper, we explore an alternative chemical computing model introduced by Qian & Winfree in 2014, the surface CRN, which uses molecules attached to a surface such that each molecule only interacts with its immediate neighbours. Expanding on the constructions in that work, we first demonstrate that surface CRNs can emulate asynchronous and synchronous deterministic cellular automata and implement continuously active Boolean logic circuits. We introduce three new techniques for enforcing synchronization within local regions, each with a different trade-off in spatial and chemical complexity. We also demonstrate that surface CRNs can manufacture complex spatial patterns from simple initial conditions and implement interesting swarm robotic behaviours using simple local rules. Throughout all example constructions of surface CRNs, we highlight the trade-off between the ability to precisely place molecules and the ability to precisely control molecular interactions. Finally, we provide a Python simulator for surface CRNs with an easy-to-use web interface, so that readers may follow along with our examples or create their own surface CRN designs.

## 1. Introduction

Skilled chemists can do many things. They can make a dizzying variety of molecules—medicines, fuels, poisons, flavouring, detergents, paints, fertilizers, explosives. They can use combinations of molecules to build materials—fabrics, plastics, glass, concrete, metals and their alloys. They can change important properties of liquids, like salinity or acidity, or even redox potential, which lets them make a battery. They can make molecules change form or colour, which gives them the ability to detect light, starch, oxygen, pH, and build air or water quality sensors. With the right salts or other dissolved compounds, they can grow beautiful crystal structures, which can be made into jewellery and pottery.

But even the most skilled chemists cannot make anything as sophisticated as life—a large collection of molecules with impressive information-processing capabilities underlying fundamental behaviours such as development, learning, and self-repair [1]. The basic components of life—lipids, nucleic acids and proteins—can all be chemically synthesized. Simple interactions between chemically synthesized molecules can carry out dynamic behaviours—oscillation or chaotic behaviour (e.g. the Bray–Liebhafsky reaction [2], the Belousov–Zhabotinsky reaction [3–6] and the Briggs–Rauscher reaction [7]). Systems including dozens to hundreds of chemically synthesized molecules, nucleic acids in particular, can be built to perform information-processing tasks including Boolean logic computation [8–10], neural network computation [11,12] and more [13,14]. Chemically synthesized molecules can also be programmed to self-assemble into structures with a variety of shapes [15–17]. However, these systems are still far less complex than what is seen in nature.

Is it possible to create much more complex chemical systems than current technologies?

Theoretically, yes. Perhaps the most well-known and well-understood formal model of chemistry is the abstract chemical reaction network (CRN), which encodes each chemical reaction in a system of chemical reactions as a mapping from a (multi)set of abstract reactant species to a (multi)set of abstract product species, along with a rate constant. CRN models differ in how these mappings are applied. Stochastic, discrete CRN models give positive integer values to molecular counts and typically treat reactions as events in a continuous-time Markov chain [18]. For very large numbers of molecules, stochastic CRNs can be approximated by continuous, deterministic mass-action CRN models, in which chemicals have non-negative, real-valued concentrations that change according to a set of ordinary differential equations determined by the CRN for the system. In both types of CRN models, molecules are well mixed—every molecule can freely float around and come into contact with every other molecule in the system.

Deterministic mass-action CRNs are Turing-universal [19], suggesting that chemistry is, in principle, as powerful as any programming language and can compute anything, even when restricted to reactions that can be understood with simplistic models. Stochastic CRNs can also compute anything, although the computation is only correct most of the time—occasionally it is wrong [20,21]. Alternatively, they can compute anything with guaranteed correctness if we could obtain the output of the computation only when time goes to infinity—otherwise it may still be wrong [22]. Conversely, some computational schemes are possible with stochastic CRNs but not with deterministic CRNs. For example, stochastic CRNs can directly represent complex probability distributions [23], perform probabilistic inference [24], and use the CRN’s inherent stochasticity to help solve combinatorial search problems [25].

Even though in theory well-mixed chemistry is sufficiently powerful for carrying out arbitrarily complex information-processing tasks, practically, it is hard to scale up the complexity beyond certain limits. For example, deterministic mass-action CRNs are Turing-universal only if concentrations can be guaranteed to have arbitrarily small relative errors, as these concentrations must encode arbitrarily precise analogue values used in the computation [19]. Stochastic CRNs have a different practical problem: the number of molecules required for a task grows exponentially with increasingly complex tasks [21].

One problem fundamental to both deterministic and stochastic CRNs is that the entire ‘program’ of a CRN is encoded in the interactions between molecules, and designing a large collection of molecules to interact with each other with specificity is, in general, difficult. It is inevitable that when two or more molecules come into contact with each other in a well-mixed system, even if they are not designed to interact with each other, unwanted side reactions will occasionally occur. A larger collection of molecules necessarily has a higher probability of side reactions, simply because there are more possibilities for every molecule to interact with every other molecule. High-order side reactions can be repressed by using smaller concentrations of chemical species—but reducing the concentrations will also slow down a CRN. Thus, there is a trade-off between the *accuracy* and *speed* of a CRN-based computation.

One possible solution to the problem of restraining undesirable interactions relies on spatial separation rather than specificity. This strategy is familiar from the world of electronic engineering: in silicon circuits, interactions between components are encoded by *spatial position*. Two AND gates, for example, do not interact unless they are physically connected by a wire, even though their design is identical and their signal carrier (electrons) is identical. Moreover, it is relatively easy to place electronic components with high accuracy, and we can leverage this power to build enormously complex devices from a remarkably small set of unique interacting component types. Biology found this solution long before engineers did. In the brain, neural circuits are largely determined by their spatial layout and connectivity; release of the same neurotransmitter yields different results depending on where it is released. Even at the subcellular level, biology exploits spatial organization to control biochemical circuit function, with scaffold proteins being a primary example [26]. Turning back to engineering efforts to program chemical systems, one could imagine taking similar advantage of spatial separation by, for example, assembling molecules into *polymers* or tethering molecules onto a *surface* so that geometry will limit which molecules can reach each other (in the best case they can only touch their immediate neighbours). This prospect has engendered a considerable body of theoretical and experimental work in DNA nanotechnology [27–36].

In the course of his explorations of reversible computing and the thermodynamics of computation, Charles Bennett exhibited a theoretical polymer CRN capable of simulating arbitrary Turing machines, with each polymer in solution acting independently and in parallel [37]. However, it remains unclear how to engineer the hypothetical enzymes needed in Bennett’s construction. Plausible implementations of Turing-universal polymer CRNs have been proposed using DNA nanotechnology, but the constructions only work when there is a single copy of certain molecules [27–29], which imposes significant technical challenges in practice and eliminates the possibility of parallelism. It is natural to expect that surface-based chemistry will have greater computational capabilities than polymer chemistry. In their 2014 paper [38], Qian and Winfree proposed a simple but powerful surface CRN model, along with a plausible implementation using DNA nanotechnology, which now allows us to explore (i) what chemistry on a surface, in principle, can do, (ii) what constraints, if any, being on a surface imposes on chemistry, and most importantly, (iii) what it is *better* at doing than well-mixed CRNs and polymer CRNs.

As it happens, chemistry on a surface can do quite a lot. Like well-mixed chemistry, even simple natural surface chemistry can encode surprisingly complex behaviour. In one striking example, administration of oxygen and carbon monoxide gasses to a well-defined platinum surface can induce bulk oscillations, spiral patterns, or turbulence, depending on the ratios of gasses used [39]. Other, somewhat more complex surface-chemistry reactions can produce Sierpiński triangles [40–42], labyrinthine mazes [43] and other complex (and switchable!) patterns [44]. That such simple systems can produce rich behaviour suggests that surface chemistry in general ought to be quite powerful.

Of course, different chemistries do different things—alloys of metals behave differently from thin-film liquids, which behave differently from proteins embedded in a membrane. The surface CRN model does not attempt to give an accounting of each and every possible kind of surface-based chemistry, but rather explore the general class of *chemical reactions between spatially constrained molecules* using a simple model. Qian & Winfree used surface CRNs to generate complex dynamic spatial patterns and showed them capable of directly emulating both Boolean logic circuits and Turing machines. We will expand on these examples and demonstrate several other things that surface CRNs can do, some of which are obvious and some not. You can follow along with our examples using our surface CRN simulator, either online or on your own computer with Python (see box 1).

Box 1. **Following along with our simulator.**

If you would like to follow along with the examples in this paper, you can do so online at http://www.dna.caltech.edu/Surface_CRN_Simulator, which presents an interface to the surface CRN simulation Python package we used for most of our design and visualization. The website has all of the examples we present, plus a few extra examples for fun. You can also define and test your own surface CRNs through the website, with the restriction that they must use a square or hex grid.

For users who prefer to run our code directly, or would like to extend it, our simulator is also available as the pip-installable Python package ‘surface_crns’. You can install it from the Python Package Index (https://pypi.org) or from our GitHub page at https://github.com/sclamons/surface_crns. This will let you watch your surface CRNs run in ‘real-time’, and lets you step through simulations one reaction at a time. If you are comfortable programming with Python, you can also use our Python package to define surface CRNs with more complex custom geometry or visualization.

Throughout this paper, many of the existing systems we reference will use DNA nanotechnology. This is because DNA is a readily programmed substrate, in the sense that it is straightforward to specify binding and reactions between arbitrary species, and so DNA nanotechnology provides, for the moment, many of the best examples of programmable chemistry [45–48]. We wish to emphasize that the surface CRN framework can just as easily apply to *any* system of molecules with programmable interactions that live on a surface. We hope that in the future, this will encompass a wide range of chemistries using a wide range of substrates.

## 2. Review: what is a surface chemical reaction network?

We follow the definition of a surface CRN as introduced in Qian & Winfree [38]: informally, it is a CRN in which individual molecules are localized to sites on a surface and can only interact with neighbouring molecules. Alternatively, a surface CRN is an asynchronous, stochastic cellular automaton with transition rules that resemble CRN reaction rules. The surface CRN model is *not* intended to replicate or model all of the possible behaviours of chemistry that happens on or near a surface; nor is the surface CRN model intended to accurately describe what is known in the field of surface chemistry. Rather, the surface CRN is a simple model of CRN-like chemistry in which molecular interactions are restricted geometrically, which we hope will clarify understanding and inspire construction of computational chemical systems on surfaces.

More formally, a surface CRN is a continuous-time Markov chain specified by a lattice *L* of connected sites *i* ∈ *L* with a state *s*_*i*_ at each site *i*, along with a set of transition rules *r* ∈ *R* of either the form *A* → *B* or *A* + *B* → *C* + *D*, each with a reaction rate *λ*_*r*_. The surface CRN model is defined for arbitrary graphs of sites, but for simplicity, we will consider only square-grid lattices in this paper (though our simulator can handle arbitrary graphs). The state at each site is always a single species, so we will often use ‘state’ and ‘species’ interchangeably. A species represents some smallest physical unit of the system (e.g. proteins covalently bonded to a glass slide, monomers in a plastic polymer, DNA complexes attached to a DNA origami sheet, or local lattice configurations in a crystal) such that local interactions can be represented as reactions. The connections between sites in the graph define which pairs of species are physically close enough to interact. Where applicable, an ‘empty’ state (e.g. *E*) may be defined for sites which do not hold anything. Note that the state of the Markov chain under this definition encompasses the entire surface, and thus the states of all sites together.

As in traditional CRNs, we refer to the species on the left-hand side of the ‘ → ’ in a reaction as reactants and the species on the right-hand side of the ‘ → ’ as products.

Unimolecular reactions (those of the form *A* → *B*) can occur at any site with a state matching the reactant. For example, the rule ON → OFF would cause any ON species to spontaneously and instantly flip to an OFF species when the reaction (stochastically) occurs. Again, a reaction rate associated with the rule determines the average frequency at which the reaction will occur.

Bimolecular reactions (those of the form *A* + *B* → *C* + *D*) can occur anywhere that the two reactant species are present and next to each other according to the connectivity of the surface on which they sit. At the moment when it occurs, a bimolecular reaction simultaneously and instantaneously transforms both reactants into products. Note that surface CRNs do not have any absolute orientation, so the absolute order of the reactants does not matter; however, the orientation of the products will match the orientation of the reactants in the lattice. Thus, the rule *A* + *B* → *X* + *Y* can occur anywhere that an *A* is next to a *B*, no matter their orientations, and will always convert *A* into *X* and *B* into *Y* (but never *A* into *Y* or *B* into *X*).

Jumps in the surface CRN process correspond to reaction events. An event is defined as a tuple (*I*, *r*, *t* + Δ*t*), where *I* is a set of one or two sites, *r* is a transition rule with the same number of sites as *I*, *t* is the time of the last event (or 0 if it is the first event), and Δ*t* is the time between the event and the previous event. At the beginning of the process and immediately after each jump event, the possible events are all (*I*, *r*, *t* + Δ*t*) for which {*s*_*i*_}, *i* ∈ *I* are exactly the reactants of *r*, with Δ*t* drawn from an exponential distribution with mean 1/*λ*_*r*_. The event with the minimum Δ*t* then occurs at time *t* + Δ*t*, at which time the reactant states at sites *I* are replaced by the products of *r*. At this point, the list of possible events is recalculated, and the process is repeated. Simulation of surface CRNs can, therefore, be considered a variant of the Gillespie algorithm [18] for stochastic chemical kinetics, and thus is susceptible to similar optimizations [49] (see box 2).

Box 2. **Efficient surface CRN simulation.**

Surface CRNs can be simulated using a variation of the Gillespie algorithm for simulating stochastic chemical reaction networks. Briefly, using variables from the text:

1. Initialize with a global state at time *t* = 0.
2. For each reaction *r* that can occur at a site or pair of sites, draw a time-to-event Δ*t* for that reaction from an exponential distribution with mean 1/*λ*_*r*_. Schedule this reaction to occur at time *t* + Δ*t*.
3. For the reaction with the smallest scheduled time, change the reactants of that reaction to its products and set *t* = *t* + Δ*t*.
4. Repeat from step 2 until a stop condition.

The naive method redundantly recalculates time-to-events for every site and pair of neighbouring sites after every reaction, with total time complexity roughly *O*(*N* × *R*), where *N* is the number of sites in the surface CRN and *R* is the total number of reaction events simulated. We instead leverage previously calculated next-reaction times by storing all possible reactions in a priority queue, sorted by the time at which each reaction will occur:

1. Initialize with a global state at time *t* = 0.
2. For each reaction that can occur, draw a time-to-event as described and add that reaction to a priority queue sorted by *t* + Δ*t*.
3. Pop the first reaction from the queue. Change reactants to products. Set *t* = *t* + Δ*t*.
4. Remove from the queue all reactions involving any of the same sites as the current reaction.
5. If any new reactions can occur that involve any of the same sites as the current reaction, add them to the queue as per step 2.
6. Repeat from step 3 until a stop condition.

Popping a reaction from the queue is an *O*(log *Q*) operation (where *Q* is the maximum number of reactions in the queue at any given time) and checking for new reactions is constant in *N*, *R* and *Q*, giving total time complexity *O*(*N* + *R* log *Q*) ignoring step 4. However, step 4 (removing outdated reactions) is linear with *Q*. Our simulation avoids the need for step 4 by storing the times when each reaction is issued and the times when each site was last changed. When an event is popped off the queue, if any of its reactants were updated between the reaction’s issue time and the current time, the reaction is scrapped and another one popped off the queue. This optimization comes at the cost of bloating the queue with outdated reactions, which can slow dequeueing somewhat.

It is worth acknowledging again that the surface CRN model is not general enough to accommodate all the complexities that may arise in real surface chemistry, such as reaction rates depending on distance in an irregular graph, or depending on higher-order neighbourhood contexts. This simplification may make it less useful as a modelling language, but at the same time it makes it more appropriate as a molecular programming language, since the requirements for a systematic general-purpose implementation will be more easily met.

Note that the surface CRN is a discrete, stochastic model, related to stochastic reaction–diffusion systems [50,51] but with several key differences. First, whereas both surface CRNs and stochastic reaction–diffusion networks consider a similarly discrete number of molecules, in reaction–diffusion systems the positions of those molecules are within a continuous space while the surface CRN model considers discrete molecules at discrete positions on the surface. Therefore, surface CRNs naturally capture exclusion effects related to macromolecular crowding, which require the introduction of extra terms in the reaction–diffusion framework [52]. Furthermore, reactions in reaction–diffusion formalisms typically have no local geometry (the relative positions of reactants and products are not specified, as if the solution is locally well mixed), whereas surface CRN reactions explicitly track which reactant becomes which product and positions are preserved. Finally, while having a diffusion constant of zero would be an anomaly in a reaction–diffusion system, in a surface CRN, molecules will by default not diffuse—diffusion must be explicitly added with a reaction such as $X+E\to \;{\;}^{k}\;E+X$, where *X* is the diffusing molecule, *E* represents an empty site and the rate constant *k* determines the rate of diffusion.

The move from bulk (deterministic) to low-count (stochastic) has a practical upside: real-world realizations of surface CRNs can be very, very small. Where a whole test tube’s worth of chemicals might be required to accurately run a bulk-reaction chemical computer, one test tube could easily hold billions or trillions of tiny surfaces, each with its own surface CRN. In short, surface CRNs ought to be aggressively parallelizable.

Importantly, as we will see in several examples, putting chemical reactions on a surface opens up an interesting trade-off between the difficulty of controlling specific molecular interactions and the difficulty of laying out components in a specific spatial arrangement. If one has the technology to position many molecules precisely on a surface, surface CRN designs with few species and reactions can accomplish complex tasks yet it will be relatively easy to design the few desired molecular interactions; conversely, if one has the technology to implement many desired molecular interactions precisely, there are other surface CRN designs that can leverage that ability to create precise, complex spatial patterns from a simple initial spatial arrangement.

Now, what exactly can a surface CRN do?

## 3. Dynamic spatial patterns

### 3.1. The chaos of asynchronicity

To begin with, surface CRNs can model many familiar chemical processes. Consider diffusion of gas particles in a two-dimensional (lattice) space. We define states *G* and *S*, for gas and space, respectively, along with the reaction *G* + *S* → *S* + *G*. When a few *G* species are speckled on a field of *S*, the gas species will diffuse about at random on the lattice, gas-like, with a diffusion rate set by the rate of the reaction *G* + *S* → *S* + *G*. With the addition of reactions that change the species identities, we have a form of stochastic reaction–diffusion system, as mentioned earlier.

**Figure 1.**
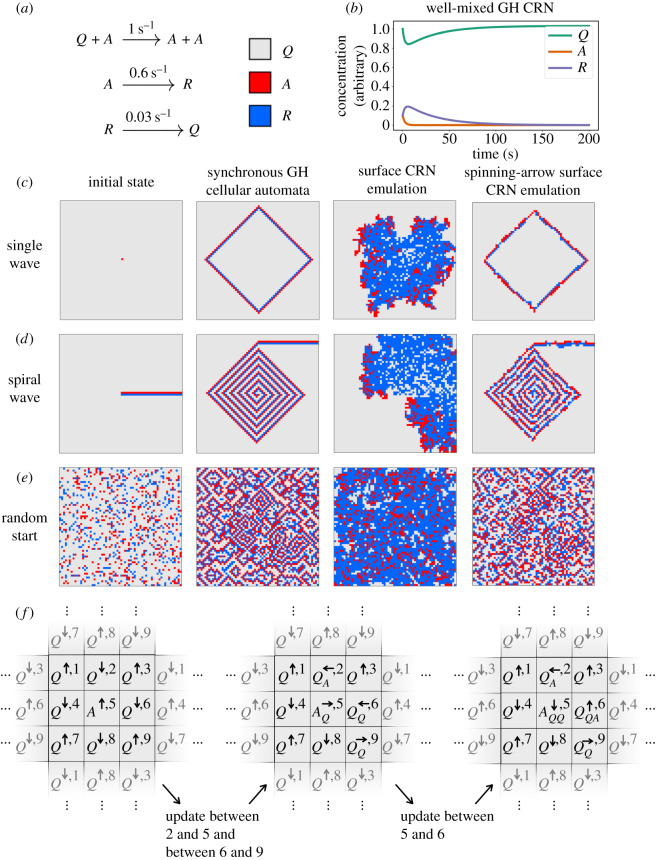
Comparison of a Greenberg–Hastings three-state excitable media cellular automaton with two surface CRN implementations. (*a*) The transition rules for the surface CRN, with colour legend for snapshots. (*b*) Behaviour of the Greenberg–Hastings system implemented as a well-mixed CRN. In bulk, this CRN displays no oscillations or other notable features. (*c*–*e*) Initial state (left column) and snapshots for the GH automaton (middle-left column) or the direct surface CRN implementation (middle-right column) or the spinning-arrow surface CRN implementation (right column). Examples are shown for a single pulse wave (*c*), a spiral wave (*d*) and a random initialization (*e*). (*f*) The spinning-arrow construction for a (locally) synchronous Greenberg–Hastings automaton using a surface CRN. Each box is a single site in the surface CRN, which emulates the function of a single cell in a synchronous automaton. At left, arrows are shown in initial positions, arranged to avoid deadlock. The middle shows the surface CRN after two updates—one between sites 2 and 5, and another between sites 6 and 9. Sites 5 and 6 are now ready to react together. At right, we see the result of the 5–6 reaction.

Surface CRNs can also model chemistry at a more coarse-grained scale. Consider the patterns generated by the Greenberg–Hastings (GH) cellular automaton [53], which is a discrete version of the beautiful and dynamic Belousov–Zhabotinsky reaction [3,6,54]. The GH cellular automaton is a discrete, synchronous, deterministic automaton where each cell has one of three states: *Q* (quiescent), *A* (active) or *R* (refractory). At each step of the automaton: any quiescent cell next to an active cell becomes active; any active cell becomes refractory; and any refractory cell becomes quiescent. Depending on the automaton’s initial configuration, it can produce single waves, infinite spirals, or complex spiral patterns (figure 1*c*–*e*, middle-left column).

The GH automaton can be modelled by a three-reaction surface CRN (figure 1*a*). This CRN has the property that if all possible reactions were examined simultaneously, and all species changes were applied simultaneously, then the behaviour of the synchronous GH cellular automaton would be reproduced exactly. However, as surface CRNs have asynchronous local updates, the GH surface CRN is stochastic and its qualitative behaviour depends on each reaction’s exact rate constant. With the rates shown in figure 1*a*, the surface CRN approximates some features of the behaviour of the GH automaton, but the waves and spirals produced in the surface CRN version of the GH system are thick and uneven, with ragged edges.

The GH surface CRN lacks the strict synchronicity of the GH automaton, which is what allows the original GH automaton to produce fine-grained, structured patterns like the spiral wave (figure 1*d*). Structures in the CRN are brittle—a clean wavefront will quickly break apart as some parts of the wave spread more rapidly than others. This causes the wavefront to break up, peter out, or double back on itself, so that a spiral that lasts forever in the synchronous GH automaton will be a temporary storm in the surface CRN. In this case, at least, asynchronicity in reactions washes out fine structure in the surface CRN.

The GH cellular automaton can be thought of not just as a specific model of the Belousov–Zhabotinsky reaction, but rather as a generic model of *excitable media* that support propagation of temporary activity through an otherwise quiescent space. Systems as diverse as metal surface oxidation, protein signalling on oocyte membranes, neural action potentials, heart muscle contractions, slime mould aggregation, lichen growth, forest fires, infectious disease outbreaks and star formation, all can exhibit spiral waves and have been studied as excitable media [55–58]. Interestingly, well-known stochastic spatial models of forest fire and infectious disease propagation correspond closely to the GH surface CRN, with the *Q*, *A* and *R* states corresponding to green, burning and burnt trees, or to susceptible, infected and resistant hosts, respectively [57,58].

Going back to the example of the GH model, we saw that an inherently asynchronous surface CRN implementation of the same local logic (and with well-tuned rate constants) can generate some similar kinds of behaviours as the original synchronous cellular automata (e.g. wave propagation), while other important features (e.g. wavefronts remaining unbroken) fail in the asynchronous surface CRN implementation. These differences can be understood in terms of conserved quantities that the synchronous updates preserve [59], but which are not preserved by the asynchronous updates (see box 3).

Box 3. **Conserved quantities of surface CRNs.**

The GH cellular automaton has a conserved property called the ‘winding number’ that is important for several proofs of its behaviour. Pick any directed, closed path through lattice sites in a GH automaton. That path will cross through zero or more ‘cycles’ of *Q* → *A* → *R* (in either direction). The net number of such cycles the path crosses (forward minus reverse) is its winding number. A simple way to compute the winding number is to traverse the path, tallying +1 for each forward step, − 1 for each backward step, and then dividing the total by 3. As an example, we write down the states along a possible path, and below them, the tallies for going from the given cell to the next cell:
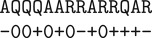

The tallies sum to 3 so the winding number is 1.

Interestingly, the winding number of any particular spatial path in a GH automaton with a fixed initial condition will remain the same as the automaton progresses, regardless of the initial configuration of the automaton or the topology of the lattice (e.g. square, hexagonal, triangular, irregular, etc.). This conserved quantity can be used to prove, for example, conditions under which a particular GH automaton will continue indefinitely or peter out to a uniform quiescent state.

— Does the surface CRN version of the GH automaton preserve winding number? Why or why not?
— Can you identify any other conserved quantities in the surface CRN GH automaton, or modify the reactions such that analogous quantities are conserved?

You may wish to revisit these exercises for the other surface CRN implementations of the GH automaton given in later sections.

More generally, it has long been known in the cellular automaton literature that asynchronous updates often destroy behaviours that were of great interest in the synchronous case [60,61]. To produce, say, the dazzlingly complex and specific phenomena of Conway’s Game of Life [62] (an example to which we will return shortly), some kind of synchronization between reactions seems necessary. Can a surface CRN, which is intrinsically asynchronous, emulate the function of a truly synchronous cellular automaton?

### 3.2. One-to-one ‘spinning-arrow’ construction of locally synchronous automata

Yes, it can, at least in the most important senses. Again, the cellular automata literature provides some guidance: many techniques have been invented whereby an asynchronous cellular automaton can, by incorporating extra states and extra rules, enforce that the information flow of the synchronous system remains intact within the asynchronous system [63–65]. However, although the surface CRN model can be seen as a subclass of asynchronous cellular automata, the fact that surface CRN updates involve just two sites at a time, and are orientation-invariant, poses significant constraints that make direct application of prior techniques impossible—but as we will show, mechanisms with a similar spirit do work.

One way for a surface CRN to emulate the function of a synchronous automaton is for each site to (a) accumulate information about all of its neighbours and (b) update itself based on that information in a way that (c) guarantees that it will not update itself before all of its neighbours have learned its state (so that its neighbours will not get stuck, unable to update, or update based on the wrong information about the cell). In general, this requires careful set-up of both the transition rules and initial conditions of the surface CRN, particularly to avoid deadlocking. Here, we will describe such a scheme for emulating any cellular automaton with a square-grid geometry and Von Neumann neighbourhood (i.e. one where each cell only considers the states of the four neighbouring cells with which it shares an edge, but not the ones that border on only a corner). The basic principle is that the identity of the state at each site, in addition to specifying the current state of the cell that it is emulating, also has a directional arrow pointing to the next site it needs information from. When two sites’ arrows point toward each other, both sites spin their arrows in opposite directions (e.g. both clockwise) and update an internal label that tracks what information that site has gathered from its neighbours. Once a site’s arrow has spun once all the way around, a set of unimolecular rules changes that site’s state according to the information it has gathered from its neighbours, resetting the arrow and preparing it for the next update. Note that care must be taken in the initial set-up of the CRN so that every site’s arrows *do* spin completely without deadlocking (see box 4).

Box 4. **Spinning-arrow deadlocks.**

The spinning-arrow emulation scheme shown in this section requires careful arrangement of initial conditions—with the wrong starting arrangement, arrows can easily become deadlocked, unable to update.

— Can you prove that the initial configuration shown in figure 1*f* will not deadlock?
— How many initial configurations are there, in terms of initial arrow direction, that avoid deadlock?
— If the surface is not infinite, then extra species and reactions are needed to mark the boundary and perform updates there that will not induce deadlock. Can you design them?
— Can you adapt the spinning-arrow emulation for surface CRNs with a hex grid graph (instead of a square grid)?

Critically, each emulated cell cannot update its state until it has received state information from all of its neighbours, and it cannot receive information from its neighbours without simultaneously sending its own state information. The result is that a cell can never be more than one ‘clock tick’ ahead of any of its neighbours, and will only send its state information when it is at the *same* ‘clock tick’ as its neighbour—it is *locally* synchronous. Note that there is no guarantee of *exact* synchronicity between distant cells in a locally synchronous automaton (although cells distance *d* from each other can be at most *d* ‘clock ticks’ apart). Nevertheless, because each cell only communicates with other cells at the same ‘clock tick’, the state history of each cell is guaranteed to match that of an equivalent cell in a globally synchronous cellular automaton. In other words, the system will behave as though it were a synchronous automaton, but with some variation in the speed at which each cell updates.

One immediate problem with the spinning-arrow scheme is that species in a surface CRN do not perceive local orientation. Sites can be next to each other or not next to each other, but reactions cannot be restricted based on reactants being ‘to the left of’ or ‘to the right of’ each other, so two arrows ‘pointing toward each other’ could just as easily be ‘pointing away from each other’ and the same reactions will apply. To introduce a notion of directionality to the scheme, we initialize the surface in a pattern with location labels at each site, dividing the grid into a repeating 3 × 3 grid (figure 1*f* ). Adding site labels allows reactions to distinguish arrows pointing together from arrows pointing apart—for example, ← at a 2 site and → at a 1 site point toward each other, while ← at site 2 and → at a site 3 point away from each other.

So, each site in the spinning-arrow emulation has a state that encodes:

— The current state of the emulated cell in the synchronous automaton.
— Local positional information within a 3 × 3 repeating grid.
— The direction of an ‘arrow’ pointing toward the next site to update from.
— A memory recording information that the site has received from neighbours during this update cycle.

More formally, consider any synchronous cellular automaton with Von Neumann neighbourhood, *k* states $\{s_{1},s_{2},\ldots ,s_{k}\}=S$, and an update rule *f*(self, *N*, *E*, *W*, *S*) where self∈S is the state of a cell and N,E,W,S∈S are the states of the cell’s northern, eastern, western and southern neighbours, respectively. Each site in the emulating surface CRN has a state of the form $X_{\text{info}}^{d,p}$, where X∈S is the state of the emulated automaton at that site, *d* ∈ { ← , ↑ , ↓ , → } is the direction of the site’s arrow, *p* ∈ {1, …, 9} is the local position of the site, and $\text{info}\in S^{*}$ is a list of collected information about the states of the northern, eastern, western and/or southern neighbours of the site that have exchanged information with the site, in an order based on the order of exchange.

State exchange is mediated by bimolecular reactions of the following form, where X∈S is an emulated state at some position *p* ∈ {1, …, 9}, *N*, *E*, *S*, *W* are the emulated states to the north, east, south and west of *p* at positions *n*, *e*, *s*, *w* ∈ {1, …, 9}, respectively:

$$\begin{array}{rl} X^{↑,p}+N^{↓,n} & \to X_{N}^{\to ,p}+N_{X}^{\leftarrow ,n}\\ X_{N}^{\to ,p}+E_{S^{\prime }}^{\leftarrow ,e} & \to X_{NE}^{↓,p}+E_{S^{\prime }X}^{↑,e}\\ X_{NE}^{↓,p}+S_{S^{\prime }W^{\prime }}^{↑,s} & \to X_{NES}^{\leftarrow ,p}+S_{S^{\prime }W^{\prime }X}^{\to ,s}\\ X_{NES}^{\leftarrow ,p}+W_{S^{\prime }W^{\prime }N^{\prime }}^{\to ,w} & \to X_{NESW}^{↑,p}+W_{S^{\prime }W^{\prime }N^{\prime }X}^{↓,w}. \end{array}$$

While these reactions at first appear to treat only a subset of possible local configurations, these are the only cases that arise if the surface CRN starts with arrows and position labels organized as shown at the left of figure 1*f* .

Finally, states are updated using unimolecular rules of the form:

$$X_{NESW}^{↑,p}\to f{\left(X,N,E,W,S\right)}^{↑,p}$$

$$X_{SWNE}^{↓,p}\to f{\left(X,N,E,W,S\right)}^{↓,p}.$$

The above reactions of all forms should be defined for all appropriate pairings of *p* ∈ {1, …, 9} and *n*, *e*, *w*, *s* ∈ {1, …, 9} and $X,N,E,W,S,N^{\prime },E^{\prime },W^{\prime },S^{\prime }\in S$.

By construction, only one reaction is ever possible at a time at each site, and this reaction choice is unchanged by updates elsewhere. Consequently, the rate of each chemical reaction can only affect the timing of the surface CRN’s evolution, not its ultimate results. Thus, for this and other similar examples, we will ignore reaction rates (or, equivalently, assume that each reaction has rate 1).

As a specific case, consider a spiral wave in a (truly) synchronous GH cellular automaton (figure 1*c*, middle-left) and in an equivalent locally synchronous surface CRN implemented as above (figure 1*c*, right). Under the right initial conditions, the spiral patterns characteristic of the fully synchronous automaton are clearly visible, but they are ‘fuzzy’ because the molecules implementing the synchronous automaton are updated inherently randomly. No site will ever get ‘too far’ ahead of its neighbours, or cause them to update in an order different from that of the synchronous version, but there is no guarantee of global synchronicity.

Although this example shows that (local) synchronicity is possible in a surface CRN, it is not a very satisfying construction. All of the logic handling memory storage, state updating, and arrow-spinning has to be hard-coded by ‘brute force’ into the species and reactions defining the surface CRN. Generally speaking, a locally synchronous spinning-arrow surface CRN representing a cellular automaton with *k* possible states requires a number of species that scales as *k*^5^ and a number of reactions that scales as *k*^8^. For example, the emulation of the synchronous GH automata uses 70 794 reaction rules, not including the boundary reactions required for finite grids. With further optimization that uses species subscripts to store only the information needed by *f* rather than the full neighbourhood state, GH can be implemented with a little over 3000 reactions. Still, this is not a user-friendly set of reaction rules.

### 3.3. Several-to-one ‘broadcast-swap-sum’ construction of locally synchronous automata

Another way to emulate a synchronous automaton with a locally synchronous surface CRN is to emulate each synchronous automaton cell with an array of multiple sites in the surface CRN. A several-to-one scheme also allows simulating cellular automata with Moore neighbourhoods (i.e. cellular automata whose cells communicate along corners as well as edges) without requiring direct communication across corners. We will also see that splitting a single cell into multiple sites allows us to separate the logic of site-to-site communication from the logic of site updating, which allows us to emulate the same automaton using dramatically fewer transition rules.

For example, consider a surface CRN implementation of Conway’s Game of Life, an automaton that uses information with a more complex update rule than the GH automaton [62]. In the Game of Life, each cell represents the state of a population in some small area. The population can take one of two values—‘alive’ (1) or ‘dead’ (0). The Game of Life automaton executes synchronously, with the new state at each position determined by its previous state and number of ‘alive’ cells among its neighbours (including cells touching by a corner) in the following way:

— Any live cell with zero or one live neighbours dies (underpopulation).
— Any live cell with two or three live neighbours remains alive (survival).
— Any live cell with four or more live neighbours dies (overpopulation).
— Any dead cell with exactly three neighbours becomes alive (colonization).
— Any other dead cell remains dead.

**Figure 2.**
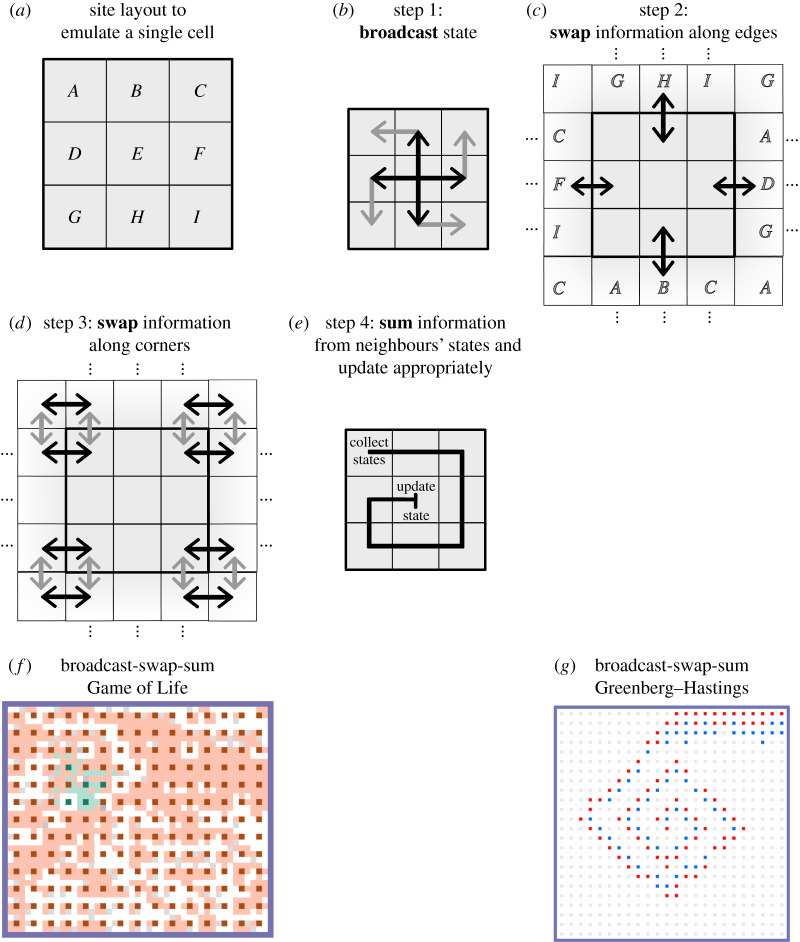
A ‘broadcast-swap-sum’ construction of locally synchronous automata. (*a*) Nine molecules emulating a single cell in a synchronous cellular automaton. The current state is held in position E, while the other molecules handle communication with neighbouring cell blocks. (*b*–*e*) A general scheme for updating such an automaton. Arrows represent information flow at different stages of the update. (*f* ) Simulation of a glider from Conway’s Game of Life, implemented with a 3 × 3 broadcast-swap-sum surface CRN emulation. Dark red sites represent ‘dead’ cells; dark green sites are ‘alive’ cells; light red and green sites are ‘wire’ sites carrying information from ‘dead’ and ‘alive’ cells, respectively; grey sites are ‘wire’ sites in the process of counting neighbouring live cells; white sites are ‘wire’ sites waiting for information from their corresponding sites; and purple sites represent an edge condition. (*g*) Simulation of a locally synchronous spiral wave using a broadcast-swap-sum emulation of a Greenberg–Hastings automaton, with simplified colours to make the spiral easier to visualize.

In a broadcast-swap-sum emulation, each cell in the Game of Life is represented by a 3 × 3 grid of sites in the surface CRN, labelled A through I from top to bottom, left to right (figure 2*a*). The centre molecule (position E) stores the current state of the Game of Life cell (0 or 1). The eight-molecule ring around E handles signal communication and processing.

We will write states in this scheme in the form $X_{i}^{S}$. X∈{A,B,…,I} indicates the position within each 3 × 3 block that the site occupies. S∈{∅,b,f,h,d,w,n,s} is a flag indicating what step of update computation the site has reached. *i* ∈ {0, 1, 2, 3, 4} is a number representing either the state of the emulated cell or the current count in a running sum of the cell’s neighbours’ states.

Update of an emulated cell begins with site E ‘transmitting’ its state to positions B, D, F and H (figure 2*b*) using the following transition rules:

$$\begin{array}{rl} E_{i}+B & \to E_{i}^{b}+B_{i}^{w}\\ E_{i}^{b}+F & \to E_{i}^{f}+F_{i}^{w}\\ E_{i}^{f}+H & \to E_{i}^{h}+H_{i}^{w}\\ E_{i}^{h}+D & \to E_{i}^{d}+D_{i}^{w}. \end{array}$$

Here, the flags *b*, *f*, *h* and *d* in the species’ names indicate the last position that was updated; *w* indicates a state that has received information from site E but that has not yet transmitted that information to the corner sites; and *i* ∈ {0, 1} is the current state of the emulated cell.

Next, similar rules transmit the emulated cell’s state from positions B, D, F, and H to positions A, G, C, and I, respectively, and set positions B, D, F and H to states *B*_*i*_, *D*_*i*_, *F*_*i*_ and *H*_*i*_, indicating that those sites are ready to transmit their current state information to neighbouring 3 × 3 blocks:

$$\begin{array}{rl} B_{i}^{w}+A & \to B_{i}+A_{i}\\ D_{i}^{w}+G & \to D_{i}+G_{i}\\ F_{i}^{w}+C & \to F_{i}+C_{i}\\ H_{i}^{w}+I & \to H_{i}+I_{i}. \end{array}$$

Now emulated cells can ‘talk’ horizontally by swapping the states in positions F and D, and vertically by swapping the states in positions B and H (figure 2*c*), using the reactions

$$F_{i}+D_{j}\to F_{j}^{n}+D_{i}^{n}$$

$$\;B_{i}+H_{j}\to B_{j}^{n}+H_{i}^{n},$$

where *i*, *j* ∈ {0, 1} are the states of each emulated cell and the *n* flag indicates that the site has received information from the neighbouring emulated cell. Concurrently, corners communicate in a similar fashion. However, since corners cannot directly communicate with one another, corner sites swap states in a two-step fashion (figure 2*d*), first swapping horizontally

$$I_{i}+G_{j}\to I_{j}^{t}+G_{i}^{t}$$

$$A_{i}+C_{j}\to A_{j}^{t}+C_{i}^{t}$$

and then vertically

$$I_{i}^{t}+C_{j}^{t}\to I_{j}^{n}+C_{i}^{n}$$

$$G_{i}^{t}+A_{j}^{t}\to G_{j}^{n}+A_{i}^{n}.$$

After swapping along sides and corners, each of the eight border sites will have an *n* flag and will contain the state of one of the emulated cell’s eight neighbours. At this point, the total number of active neighbours is summed in a circular fashion (figure 2*e*) using reactions of the form

$$\begin{array}{rl} & A_{x}^{n}+B_{i}^{n}\to A+B_{\min\left(x+i,4\right)}^{s}\\ & B_{x}^{s}+C_{i}^{n}\to B+C_{\min\left(x+i,4\right)}^{s}\\ & C_{x}^{s}+F_{i}^{n}\to C+F_{\min\left(x+i,4\right)}^{s}\\ & \;F_{x}^{s}+I_{i}^{n}\to F+I_{\min\left(x+i,4\right)}^{s}\\ & \;I_{x}^{s}+H_{i}^{n}\to I+H_{\min\left(x+i,4\right)}^{s}\\ & \;H_{x}^{s}+G_{i}^{n}\to H+G_{\min\left(x+i,4\right)}^{s}\\ & \;G_{x}^{s}+D_{i}^{n}\to G+D_{\min\left(x+i,4\right)}^{s}. \end{array}$$

Here, *x* is a running sum of the number of neighbouring emulated cells with state 1, which is capped at 4 for compression since the behaviour of a cell of the Game of Life does not change past four neighbouring ‘alive’ cells. The *s* flag indicates that the site currently holds the running sum for the emulated cell. This set of reactions also resets the ring and prepares it for the next round.

Finally, the sum in position D is combined with the current state, setting the new state and resetting D, by a set of rules of the form $D_{x}^{s}+E_{i}^{d}\to D+E_{G(x,i)}$, where *G*(*x*, *i*) is the update rule for the Game of Life—that is, 1 if *x* = 3 or if *i* = 1 and *x* = 2, otherwise 0. An example of a glider from the Game of Life is shown in figure 2*f* (dark green centre squares).

A similar construction can be used to make a locally synchronous GH automaton with a broadcast-swap-sum emulation (figure 2*g*). This same strategy can be used to emulate any Moore-neighbourhood totalistic synchronous cellular automaton (i.e. those automatons for which the update rule depends only on the current state of a cell and the *total* number of neighbours with some state), or, with a more complex neighbour-summarization procedure (which would require more reactions and chemical species, but not more physical space), any Moore-neighbourhood non-totalistic cellular automaton.

Altogether, including reactions to handle boundary conditions, the broadcast-swap-sum Game of Life automaton requires 132 rules, and nine sites for each Game of Life cell, and the broadcast-swap-sum GH automaton requires only 102 reactions and 104 states—a considerable improvement over the spinning-arrow emulation technique, at least in terms of number of transition rules required to implement the behaviour. This efficiency of transition rules (which we might expect to be of prime importance in any laboratory implementation of a surface CRN) comes at two costs—larger surface CRN area per area of emulated cellular automaton, and more reaction steps per cellular automaton update.

## 4. Continuously active logic circuits

A properly constructed Game of Life of sufficient size can either simulate a Turing machine [66] or a Boolean circuit using streams of gliders to represent data lines [67]. Since the surface CRN formalism can emulate the Game of Life, it is therefore Turing-complete, which means that, in principle, surface chemistry ought to be capable of performing any computation accessible to a digital computer.

If desired, one could program a Turing machine directly as a surface CRN, as shown by Qian & Winfree [38]. In the same paper, those authors also provide a scheme for implementing arbitrary feed-forward Boolean logic circuits. These circuits function similarly to electronic logic circuits, with a few notable differences, as you will see below. A strength of Boolean logic circuits is that they can compute in parallel, allowing them to make decisions faster than equivalent Turing machines. We will show some interesting examples of circuits made using the strategy described in [38], updated slightly to accommodate feedback circuits in cases where the original strategy could produce permanent ‘traffic jams’ at wire crosses. (Also cf. [65,68] for Boolean circuits implemented as cellular automata.)

**Figure 3.**
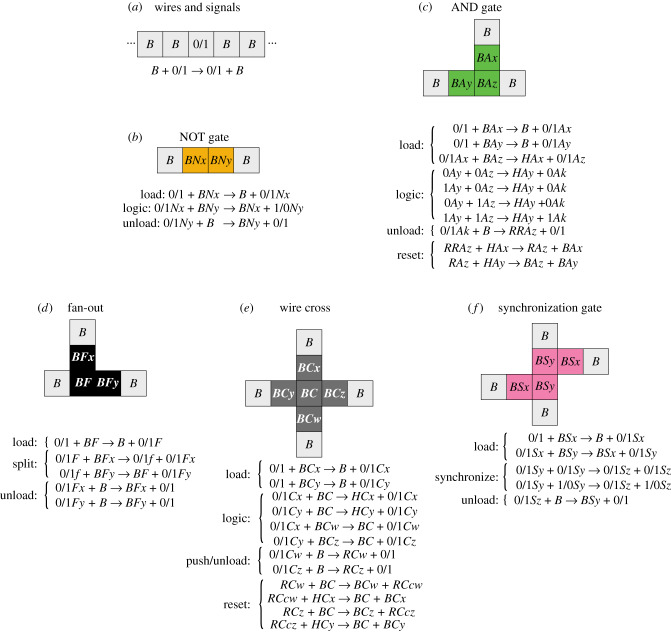
Continuously active logic circuits. *B* represents a blank wire, and 0 and 1 are states representing data packets. Gate locations use states with a three-symbol name, where the first symbol denotes the data at that position (either blank, 0, or 1), the second symbol distinguishes different gates and the third symbol distinguishes different parts of the gate. (*a*) Wires, signals and signal diffusion, (*b*) NOT gate, (*c*) AND gate, (*d*) wire fan-out, (*e*) crossing wires and (*f*) synchronization gate implemented with surface CRNs.

To review, our Boolean logic circuits are based around signal states 0 and 1, which diffuse along linear paths of wire sites *B* (blank wire) that are embedded in a field of inert states *I* (figure 3*a*). Information thus flows as discrete packets that perform a random walk along wires using two diffusion reactions 0 + *B* → *B* + 0 and 1 + *B* → *B* + 1. For the sake of brevity, we will write this pair of reactions as 0/1 + *B* → *B* + 0/1. We will continue to use this notational convention—any rule written with a ‘/’ represents two logically related reactions, compressed for space.

To compute, we use logic gates constructed from several adjacent sites representing input and output positions. The simplest gate we construct is the NOT gate, shown in figure 3*b*. The NOT gate is constructed from several states of the form *SNL*, where *S* ∈ {*B*, 0, 1} represents the data currently held at this position (either blank, 0, or 1), *N* designates the state as belonging to a NOT gate and *L* ∈ {*x*, *y*} distinguishes the input and output locations. This gate uses three sets of reactions—one to load incoming data packets (either ‘0’ or ‘1’) from a wire onto the front half of the gate, one to invert those packets and move them to the back half of the gate, and one to unload the inverted packet from the back half of the gate to another wire. For example, when the front half of a NOT gate is in a waiting state *BNx* and is next to an incoming 0 packet, the rule 0 + *BNx* → *B* + 0*Nx* loads the zero signal onto the front of the NOT gate and removes it from the wire. Similarly, a 1 can be loaded using the reaction 1 + *BNx* → *B* + 1*Nx*. Note that unlike wires, which allow bidirectional information flow, NOT gates only allow signals to pass in one direction, causing computation to ratchet forward.

A two-input logic gate can be built with an architecture similar to that of the NOT gate. A 14-reaction AND gate is shown in figure 3*c*. Four rules load incoming signals onto the *Ax* and *Ay* positions. Two more rules move the *Ax* signal onto the *Az* position, putting the *Ax* position into a holding state (*HAx*). By combining the signals from the *Ax* and *Ay* lines, four more rules calculate the correct output signal (now on the *Az* position). The output is then unloaded onto a wire, leaving the *Az* position in a reset state *RRAz*, which triggers two reactions that reset the gate and prepare it for new incoming signals. The output signal can now diffuse away, but cannot be re-loaded into the gate. Note that the AND logic occurs entirely in the four rules that combine the *Ax* and *Ay* signals—the gate can be changed to implement other logic simply by changing these four reactions. We will use OR and XOR gates in later examples built in this way.

So far, a single wire in this implementation cannot feed a signal to more than one gate (in parallel), as each data packet (0 or 1) is consumed when it is detected by a gate. To make circuits where one line can feed to multiple gate inputs, we will need an explicit fan-out mechanism. A gate implementing a two-output fan-out is shown in figure 3*d*. It uses reactions similar to those of the AND gate but with opposite flow—two rules read a signal into the centre of the gate, four rules ‘split’ the signal into the two output positions, and four more rules unload each output position’s signal onto its respective wire. A three-output fan-out can be built in a similar fashion using 14 reactions.

Finally, in order to build circuits of any reasonable complexity on a two-dimensional grid, we will need a mechanism that allows wires to cross without mixing up their signals. Figure 3*e* shows an example wire cross ‘gate’. For each axis (horizontal or vertical), the wire cross requires two rules to load a signal on the front end, two rules to push the signal to the centre (labelled so the gate ‘knows’ whether the signal is coming from *Cx* or *Cy*), two rules to push the signal to an output gate, two rules to unload the signal onto a wire, and two rules to send a reset signal to the front of the gate to prepare it for the next input.

Note that here (unlike in the logic gates shown earlier), holding and resetting the input lines is important for guaranteed wire cross function, at least for use in recurrent circuits. A circuit can be laid out such that both of the wires of the wire cross feed the same two-input gate. In such a case, a wire cross (such as the one presented in [38]) without gate-locking or similar precautions can become permanently jammed if enough inputs arrive from one line to fill up the wire cross, blocking the inputs required to allow the backed-up line to proceed.

Finally, a gate that is not strictly necessary, but that will be helpful for a later construction, is what we term a ‘synchronization gate’ (or ‘sync gate’). This gate is essentially two linked repeater gates (NOT gates with the inversion logic removed) that wait until both inputs are present before sending them on their respective journeys, which can be used to ensure synchronization between different parts of a circuit. This gate could instead be built purely using the other logic gates already shown above, at the cost of clarity.

**Figure 4.**
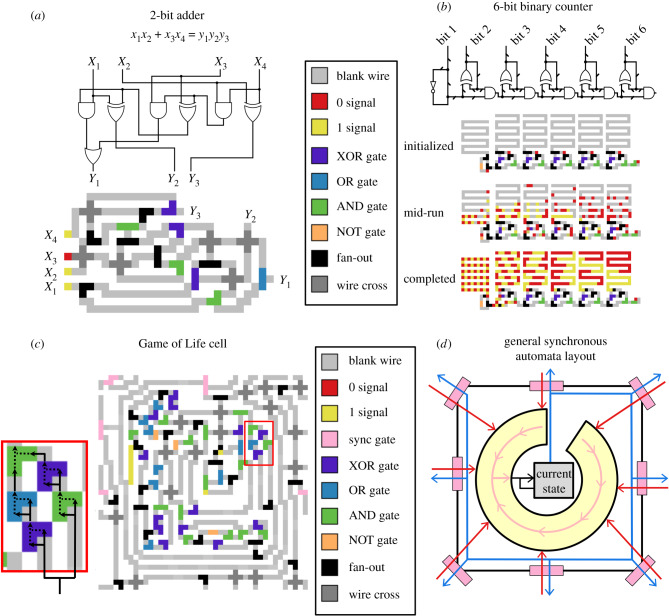
Example logic circuits and a logic circuit emulation for locally synchronous cellular automata. (*a*–*c*) Example circuits built using the rules and gates from figure 3, plus a synchronization gate. Inset in (*c*) highlights the use of ‘sequential gates’ whose two inputs are sequential signals on the same input line, which are useful for efficiently computing certain Boolean statements with repeated structure. Arrows show the direction of logic flow through this motif, which outputs ‘1’ if and only if exactly three out of four sequential inputs are ‘1’ s. (*d*) General scheme for building locally synchronous cellular automata using CRNs on a surface. Blue arrows represent outgoing state information, red arrows represent incoming state information, and pink arrows represent information flow through a ‘logic ring’ that computes the next cell state. Pink rectangles are synchronization gates, which allow signals to pass only when both inputs are present.

Given these reactions and gate layouts, we can compose circuit elements and scale up to larger circuits by simply laying out signals in different initial conditions on the surface. An example surface CRN circuit that adds two 2-bit numbers is shown in figure 4*a*. Input signals (0 or 1) are initiated at *x*_1_, …, *x*_4_. These signals could be set manually during construction of the circuit, or they could be fed from an upstream source (perhaps another circuit). Signals asynchronously propagate through the circuit, with computations analogous to those in electronic circuits performed at each gate. The speed and exact timing of each reaction is random, but the eventual output of the circuit is guaranteed to be correct.

Another example that takes advantage of the continuous and reusable nature of logic circuits on a surface CRN is a 6-bit binary counter shown in its initial condition, after several rounds of output, and in its final state (figure 4*b*). At the top of this circuit, a signal diffuses around a ring with a NOT gate (which latches diffusion to one direction), switching back and forth between 0 and 1. This signal is duplicated and passed through a series of sequential half-adder circuits, where each half-adder’s inputs are its last computed value and the value computed by the previous half-adder. To make the counter’s function clearer, a wire at the output of each half-adder stores the output history of that adder, with positions farther down the wire holding older values than positions closer to the half-adder. The output can be read as a series of binary numbers, where the *n*th packet (starting from the end) of the *m*th output line is the *m*th bit of the *n*th number. These outputs could be hooked to the input of another circuit to ‘clock’ that circuit, or the counter could be used to create repeating spatial patterns.

Just as we can emulate logic circuits in a Game of Life, we can emulate the Game of Life using feedback logic circuits. Figure 4*c* shows a single cell from a Game of Life, implemented with a roughly 40 × 40 surface CRN. For scale, if such a cell were implemented on the surface of a metal crystal, with a single site at each atom, it would be order of magnitude 10 nm square; if instead it were implemented using the existing DNA origami-based scheme in [38], with origami tiles similar to those in [69], it would be approximately 200 nm across, or between 1/10 to 1/5 the length of *E. coli*, with each cell’s circuit layout covering a 3 × 3 array of origami tiles.

Each cell consists of: a core loop in the centre of the device that holds the cell’s current state (0 or 1); an outer loop that transmits a copy of the cell’s current state to each of its neighbours; and a ring-shaped block of logic that decides what the core loop’s next state should be based on the values of the cell’s neighbours. This basic structure, outlined in figure 4*d*, is general for simulating any synchronous cellular automata with local update rules computable by a logic circuit, with different circuits in the logic ring yielding different automata.

At the beginning of each generation update, the state in the core loop is duplicated and sent (a) to the outer loop for transmission, and (b) back to the core loop for storage and computation of the next cycle state. The split signal runs clockwise around the outer ring starting from the top. At each border with another cell, a copy of the state signal is created and waits until a matching signal is received from the neighbour. Synchronization gates (see figure 3*f*) at these junctions ensure that no cell can update more than one step ahead of its neighbours. Once the neighbour’s signal is received, it is fed into the logic ring, where a circuit in the logic ring determines what the cell’s next state should be. Finally, the result of that computation is fed into the core loop, replacing the previous value, and the cycle begins again.

The Game of Life update calculation involves detecting whether or not exactly three or four out of nine neighbours are ‘alive’ (pass a ‘1’ signal), which is somewhat cumbersome to check using a Boolean logic circuit. To keep this circuit to a reasonable size, we take advantage of the digital, sequential nature of surface CRN logic circuits to make ‘sequential’ logic gates that act on two sequential signals from the same input line, rather than two parallel inputs on separate input lines. For example, the inset shown in figure 4*c* shows a five-gate motif that produces output with every four consecutive signals it receives, yielding ‘1’ if exactly three of those signals are ‘1’s and ‘0’ otherwise. An equivalent standard Boolean logic circuit would require two additional logic gates and approximately five wire crosses and four fan-outs. Note that sequential logic gates use exactly the same states and transition rules as a standard surface CRN logic gate, only arranged differently on the surface.

At first glance, this implementation of the Game of Life seems cumbersome compared to the much more compact emulation method described in §3. Using logic circuits certainly requires more surface area and more running time than directly emulating the same Game of Life. However, the circuit-based implementation uses 110 reactions as written (including synchronization gates and ‘repeater gates’ that serve only to speed computation; see the Game of Life examples on our website for details). This number can be reduced to 46 by removing redundant gates and re-using rules for data-loading using a common input state, bringing the total reaction rule set down to less than a third the number required for the broadcast-swap-sum Game of Life emulation (see box 5).

Box 5. **Efficient logic circuits.**

Wires, signal diffusion, two- and three-output fan-out, wire-crossing, synchronization and repeater gates, NOT, OR, XOR and NOT gates—as we have done here—requires a total of 110 transition rules. That is a lot of molecular interactions, from the point of view of an experimenter trying to physically build such a circuit. We can shrink this rule set considerably, sacrificing only spatial compactness and readability of the final circuit design, by removing repeater, sync, AND, OR, XOR and NOT gates (as these can be emulated with only NOR gates), and by eliminating the three-output fan-out gate. This brings the total number of reactions required for logic down to 46 transition rules and about as many unique species. Can you do better?

— What is the smallest rule set you can come up with that still allows you to build continuously active recurrent logic circuits?
— What is the smallest number of distinct species for which it is still possible to create a rule set that allows you to build continuously active recurrent logic circuits?
— Consider a surface CRN with a limited number of states available at each site. One might, for example, build a surface CRN with many different species at *different* sites, but in which each single site may only flip between N different species. Our logic circuit implementation requires up to seven species at any single site. What is the minimum number of species per site you need for logic? Can you do it with three? With two?
— Can you build Boolean logic circuits using only reversible reactions? (Hint: you can.) What would drive these gates forward?

Moreover, the same strategy can be used to construct any synchronous cellular automata using exactly the same rule set, varying only the initial layout of species on the surface. This is a considerable advantage in an experimental setting, as only one set of on-surface reactions need be designed and validated, along with the ability to lay down arbitrary initial surface layouts, in order to build any molecularly implemented (synchronous or asynchronous!) cellular automata, recurrent digital circuit, or Turing machine. In principle, even a fully functional microprocessor [70] could be implemented using exactly the same surface CRN reactions shown here. (Implementing universal circuit constructions with reversible surface CRN reactions also establishes connections to the physics of computation [71].)

This ease of scaling contrasts with existing programmable molecular implementations of logic using, for example, DNA complexes in a well-mixed test tube or protein in a cell. In those schemes, each wire is encoded with a different species, and each new gate requires the design of a set of chemical reactions with new molecular species.

Of course, the benefits of surface CRNs are not free, and this example is the first that clearly illustrates the design trade-off alluded to at the end of §2—in this case, surface CRN-based logic circuits trade difficulty in molecular *interaction design* for difficulty of molecular *placement*. If you want to build a large logic circuit by implementing a surface CRN, you must be able to precisely position a large number of molecules into the correct initial state; in return, you can build circuits of arbitrary size with a relatively small, fixed number of molecular interaction designs.

## 5. Manufacturing

We have now seen that a major advantage of performing chemistry on a surface is that you can exploit power in the form of *spatial arrangement and placement* of molecules in order to reduce the complexity of desired *molecular interactions* for accomplishing the same task. We can also invert this paradigm to do the opposite—harness the ability to specify and implement precise and diverse *molecular interactions* in order to achieve complex *spatial arrangement and placement* from simple initial conditions.

Readers of a certain bent may have noticed that the 6-bit binary counter presented in §4 generated highly specific spatial patterns—in this case, alternating stripes of various lengths. The binary counter circuit can generate an arbitrarily long stream of patterned bits, which, given empty space to diffuse into, will create arbitrarily large striped patterns. What other patterns can we spontaneously generate using surface CRNs? Can surface CRNs be used to manufacture complex patterns from simpler initial conditions? The answers to these questions have clear relevance to molecular manufacturing.

**Figure 5.**
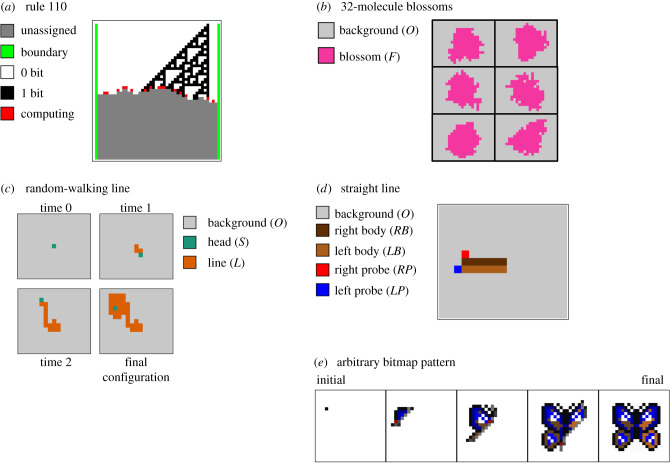
Several examples of pattern manufacturing. (*a*) Stephen Wolfram’s rule 110 elementary cellular automata, in mid-construction. In each row, emulated cells use transition rules to exchange state information, then update the state of the cell below them according to rule 110. See the ‘Elementary Cellular Automata Rule 110’ example on our online simulator for details. (*b*) Six final configurations of an irregular blossom of fixed size, each produced from an identical single-molecule seed on a uniform background. The exact pattern of the blossom is stochastic, but it will always have exactly 32 area. (*c*) Construction of a random-walking line with the rule *S* + *O* → *L* + *S* at four times. At the final configuration, this surface CRN is stuck. (*d*) Construction of a straight line. Note that in this global state, there are no sites in an *RH* or *LH* state. (*e*) Construction of an arbitrary bitmap pattern from a single starting seed against a uniform background.

We have already shown that the space of surface CRNs effectively contains all cellular automata, so patterns producible by a cellular automata ought to be producible by a surface CRN as well. For example, we can make spirals with a surface CRN GH excitable media. Any of Stephen Wolfram’s one-dimensional elementary cellular automata, along with their time histories [72], can be generated using a rule set similar to, but much more compact than, the spinning-arrow class of synchronous cellular automata (figure 5*a*). This immediately allows the construction of striping, aperiodic chaos, tree structures and Sierpiński triangles, and much more (see rules 184, 30, 90 and 110, respectively). There is a rich history of using cellular automata to model biological pattern formation and morphogenesis [73,74] so in that sense it is natural to expect that complex and useful structures can be built by surface CRNs as well.

What about specific shapes that do not correspond easily to known automata, or that might be generated more easily with a direct surface CRN implementation? We can start with a very simple manufacturing example—construction of an irregular ‘blossom’ of fixed, arbitrary size. We begin with a field of blank species (*O*), on which we add a single seed state (*S*). Here is a set of rules that converts the initial seed into an irregular blossom with area exactly 32 (figure 5*b*)

$$\begin{array}{rl} S+O & \to 4+4\\ 4+O & \to 3+3\\ 3+O & \to 2+2\\ 2+O & \to 1+1\\ 1+O & \to F+F\\ 4+F & \to F+4\\ 3+F & \to F+3\\ 2+F & \to F+2\\ 1+F & \to F+1. \end{array}$$

The seed generates two ‘4’ states, which each decay into two ‘3’ states, which continue to decay in a similar fashion until a final state (*F*). Five rules are required to encode the splitting behaviour, and four diffusion rules prevent intermediate numbered states from becoming stuck in a sea of *F*s. From a single seed state (say, a detection event by some molecular sensor), we obtain a large irregular shape with a defined size (say, a large fluorescent dot visible under a microscope or by eye). With two more rules, we can double the area of the blossom; in general, the number of rules required scales with the log of the size of the blossom.

This example has the general quality of creating something large from something small and easy to initialize, but the blossoms are not particularly complex, nor particularly specific. What about a more structured structure?

One such example is an infinite straight line, again starting from a single seed *S* against a field of open space states *O*. This exercise is worth trying before you read our solution! It can be done, and relatively simply. As a warm-up exercise, note that drawing a random one-dimensional curve is almost trivial—we can do it with a single reaction *S* + *O* → *L* + *S*. This curve will random-walk around the surface until it loops on itself and gets stuck (figure 5*c*).

It is less obvious, however, how to build a *straight* line on a surface CRN. As with the spinning-arrow construction of synchronous cellular automata, here we must contend with the fact that the surface has no built-in orientation, no structure that distinguishes left from right, top from bottom, straight from sideways. Our line will have to create such a structure. One way to do this is to make the line two molecules thick, with different states on the left and right sides. A reaction, *S* + *O* → *RH* + *LH* breaks the symmetry of the background and sets the line in motion. The two head states (reversibly) extrude probes, which look for the forward direction:

$$\begin{array}{rl} RH+O & \to RB+RP\\ RB+RP & \to RH+O\\ LH+O & \to LB+LP\\ LB+LP & \to LH+O \end{array}$$

The only places where the probes can be extended such that they touch is in front of the growing line; when they are next to each other, the two probes can convert themselves into a new head with the reaction *RP* + *LP* → *RH* + *LH*, which can then extrude its own probes and continue extending the line indefinitely. Note that the initial direction in which the line grows is random, determined stochastically by the first reaction and the first probe-connecting growth move, but once growth begins, it will continue indefinitely in a straight line (figure 5*d*).

A single line extending infinitely in a random direction is admittedly not, in itself, a particularly compelling shape. However, a line can be used as a manufacturing primitive in the construction of larger, more complex shapes, like a square or triangle, which could themselves be combined into yet-more-complex shapes, perhaps eventually forming a house, or a factory. The reader might enjoy trying these and other more complex manufacturing challenges and exploring their connection to computability via the Busy Beaver problem [75] (see box 6).

Box 6. **Manufacturing challenges.**

It is safe to say that the field of surface CRN manufacturing is a young one, and many challenges remain. Here are three for you to tackle:

— Our line-growing construction relies very much on an underlying square-grid geometry. It will not, for example, work as-written on a hexagonal grid. Can you design a version that does?
— Can you build a CRN that constructs, from an initial seed, a straight line of a specific length, rather than an infinite line? Can you do it in a way such that the number of rules required scales less than linearly with the length of the line? What about a square?
— Consider the Busy Beaver problem in computer science: what is the largest finite number of 1s that can be generated using a Turing machine with a given number of states and symbols, starting with a blank tape? Similarly, we can consider the surface CRN Busy Beaver problem: given integers *R* and *K*, define $BB_{\mathrm{CRN}}^{2D}(R,K)$ to be the largest finite, fixed number of 1s that can be reliably produced by every execution of some surface CRN with *R* reactions and *K* species, starting from a single *S* in a sea of *O* on a 2D square grid. Here, ‘reliably produced’ means that there are a finite number of reachable states, one or more of which are terminal in the sense that no further reactions are possible, every reachable state can reach a terminal state, and every terminal state has the same number of 1s (in any arrangement, and with any other species also present). The ‘blossom’ construction, for example, already provides a set of lower bounds $BB_{\mathrm{CRN}}^{2D}(2n-1,n+2)\geq 2^{n}$. Can you do better? You might want to start with the similarly defined $BB_{\mathrm{CRN}}^{1D}(R,K)$ that is confined to a 1D line of states. Or, to illuminate the influence of geometry, you might want to compare and contrast to the ‘zero-dimensional’ well-mixed case, where *BB*_CRN_(*R*, *K*) concerns a stochastic CRN starting with a single *S*.

As an extreme example of construction-by-reaction, it is possible to extend the line-creation example to create a surface CRN that builds an arbitrary bitmap pattern around a single-site seed against an otherwise-uniform background (figure 5*e*). In our implementation, a seed picks a random direction in which to extend a two-pixel-wide line in which each pixel has a unique address (i.e. there are unique species for each position), forming one edge of the image. A second, orthogonal two-pixel-wide line with unique addressing forms a second edge of the image. Then a series of reactions fill in the image starting from the corners, again with unique addressing. Finally, one reaction for each pixel converts the addressed site to a coloured final state. This algorithm requires a fairly large number of reactions, which scale linearly with the number of painted pixels. Our construction could be easily modified to simulate tile self-assembly models [76], in which case more efficient encoding could be used for some patterns that are algorithmically compressible [77]. The ability of surface CRNs to iteratively rewrite information in place, which is not possible in tile self-assembly, ought to allow more advanced constructions that provide more effective compression as well as self-healing capabilities—as has been seen in other cellular automaton models for morphogenesis [74].

Again, we wish to highlight the nature of the trade-off in surface CRN design between the required number of molecular interactions and the required number of precisely placed molecules. If you can already place molecules on a surface with high precision, then you do not need complex manufacturing algorithms of the kind shown in this section, and you will probably want to leverage surface CRNs in other ways (perhaps the ones outlined in earlier sections). If you *cannot* place molecules on a surface with high precision, but you *can* implement many desired reactions between molecules, then you can use a surface CRN to build complex designs from simple initial conditions—even, given enough reactions, arbitrarily complex patterns from a single randomly placed seed (figure 5*e*). Either way, surface CRNs can be a helpful abstraction, but they perform different work depending on what technology you already have available.

## 6. Robots and swarms

If we wish to construct complex patterns from stochastic reactions, we would do well to take inspiration from nature. For natural examples of complex construction, we need only look to ant and termite colonies. Termites, for example, begin to construct their nests using a simple set of stochastic behaviours: walk about at random; eventually pick up a mudball; wander with the mudball and eventually put it down, preferentially dropping it where there are other mudballs. A simple set of rules of this sort (usually supplemented by pheromone-laying behaviour) is sufficient for a colony of termites to collectively build piles, then towers, and eventually complex nest structures [78].

**Figure 6.**
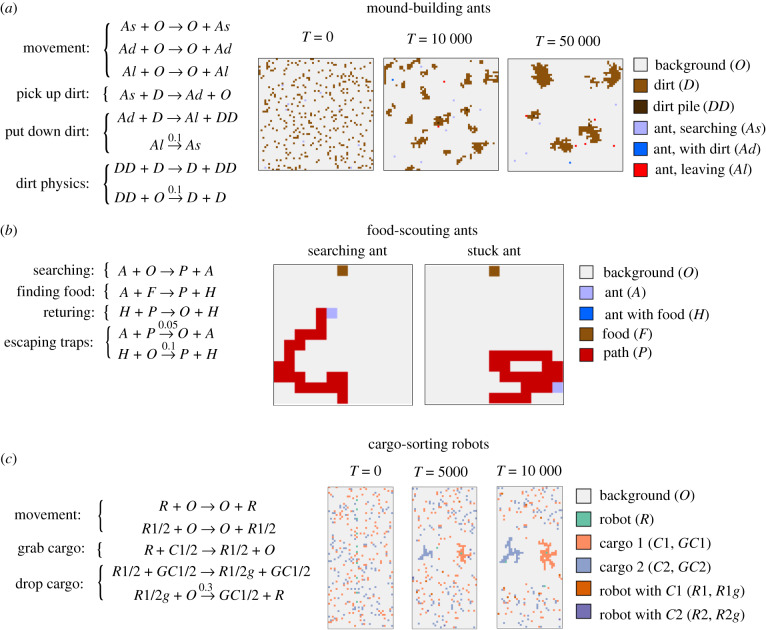
Surface CRNs with ant-like behaviour. (*a*) A swarm of ants (lavender, blue, red) collecting dirt (brown) into small piles. Unlabelled reactions have rates of 1 s^−1^. (*b*) An ant (lavender) scouting for food (brown), leaving a trail (red) to lead itself back home when it finds the food. This ant is capable of finding food and returning to its starting position, but it is not particularly robust—it will sometimes hem itself in with its own trail, trapping itself in a ring that can be difficult to escape. (*c*) A swarm of cargo-sorting robots (green) sorting two different molecular cargoes (blue and orange) into separate piles. In the initial state, there is exactly one *GC*1 site and one *GC*2 site.

While recognizing that ants are not termites, out of deference to Langton’s ant [79] and the overall superior charisma of ants over termites, we shall use a simple set of rules inspired by the nest-building behaviour of termites to build rudimentary piles using a molecular ‘ant swarm’, as shown in figure 6*a*. We begin with dirt species scattered randomly and uniformly on a field of open spaces. We place ants on this resource-rich field, and allow the ants to diffuse at random using rules similar to those used at the beginning of §3—let us call this ‘searching’. The ants can pick up dirt, transforming into an ‘ant-with-dirt’ species. Dirt-laden ants diffuse about randomly as well until they hit another dirt, at which point they deposit the dirt on the pile, forming a stacked-dirt species. A simple two-rule dirt physics allows any stacked dirt to diffuse around the dirt pile until it ‘falls off’ onto an empty space (not unlike simple sandpile physics [80]). This dirt physics helps the mounds develop a more round shape. Ants that have just put down dirt enter a temporary ‘leaving’ state in which they cannot pick-up dirt, so that they diffuse slightly away from the pile they just added to before they can pick up additional dirt.

While an observer could hardly mistake the products of this series of reactions for the complex nests of real ants, our surface CRN mound-builders are at least capable of clustering scattered dirt particles into rudimentary piles. Furthermore, they do so with considerably less underlying complexity than a real ant, and are far smaller: using molecular implementations of surface CRNs along the same lines as those in [38], our surface CRN ants act on a roughly 10^6^-fold smaller length scale, with about 10^18^-fold less mass.

Ants, of course, do much more than build small piles of dirt. Collective behaviours in animals and insects—ants included—have inspired a remarkable variety of algorithms for swarm robotics and distributed computation [81–83]. So what other ant-like behaviours might we mimic with a surface CRN?

What about scouting for food and bringing it back to the nest? We can make an extremely simple (and extremely unintelligent) food-scouting ant (*A*) that diffuses about randomly, converting open spaces (*O*) in its wake to pheromone species *P*, with the reaction *A* + *O* → *P* + *A*. When the ant finds food, it picks up the food and converts to a ‘food-bearing ant’ state *H* with the reaction *A* + *F* → *P* + *H*. A food-bearing ant walks back down its pheromone trail with the reaction *H* + *P* → *O* + *H*, converting it back into open space, until it reaches its home (figure 6*b*). This simple ant works … so long as it does not loop back on itself and become stuck. Adding two probabilistic reactions can occasionally help the ant escape such traps. To be more effective, we can improve the ant scout’s performance by adding ‘rails’ to the side of its trail to prevent the trail from overlapping with itself, and by adding a very slow reaction that causes the ant to spontaneously run back to the nest, destroying its trail along the way, so that it can unstick itself from loops (see the ‘Smarter Scouting Ant’ example on our simulator webpage for implementation details). The problem of defining and implementing a better search algorithm for molecules on a surface is an open one.

The food-finding ant example bears some resemblance to an existing class of programmable surface chemistry—surface-based molecular robotics. Currently, molecular robots can traverse pre-laid tracks or open landscapes [84–87]. Surface-based DNA robots can also sort initially dispersed cargo into separate homogeneous piles [88]. Simple molecular robots that do little more than walk on a DNA origami surface can take steps as often as once per second [89], raising the hope that more complex molecular robots need not be slow. Surface CRNs are a natural model for DNA walkers and similar molecular robots, and provide a natural format for designing and testing molecular robot algorithms.

Figure 6*c* shows an algorithm for a molecular robot (*R*) that diffuses around a field of open sites (*O*) searching for two types of cargo (*C*1 and *C*2), which it carries back to two goal sites (*GC*1 and *GC*2, respectively). This algorithm bears close resemblance to the ant mound-building algorithm at the beginning of this section. The primary difference between the two is that the cargo-sorting robot distinguishes between a cargo and its goal, and can mark dropped cargo as belonging to the goal. Because these robots drop the cargo as soon as they encounter a goal, the resulting piles develop shapes similar to diffusion-limited aggregation [90]. Whether this is realistic for a molecular robot will depend on the implementation of that robot and the nature of its cargo. An alternative formulation, closer to [88], could use specially marked ‘destination’ sites that are replaced by the cargo.

We may note at this point that a common pattern with surface CRN swarm robots is that they all must contend with the extremely limited local information available to each species. A molecule on a surface can tell if it is next to, for example, a ‘dirt’ molecule, but it has no way to directly measure whether that dirt is part of a larger pile, or how big that pile is—information which an ant brain can, presumably, compute from a number of non-local sensory cues from its eyes, limbs, olfactory system, and antennae.

Difficulties of extreme information locality can be viewed as an inherent *limitation* of surface CRN chemistry, which requires extra design effort to circumvent. Viewed another way, these difficulties are clarifying. If you are a molecule, or even an assemblage of molecules as staggeringly complex as, say, a bacteria, you do not necessarily have immediate access to ‘obvious’ information like which direction is forward, or how big a nearby object is. These are real limitations on molecular systems, and molecular machines must either work without such information, or dedicate resources to computing it.

**Figure 7.**
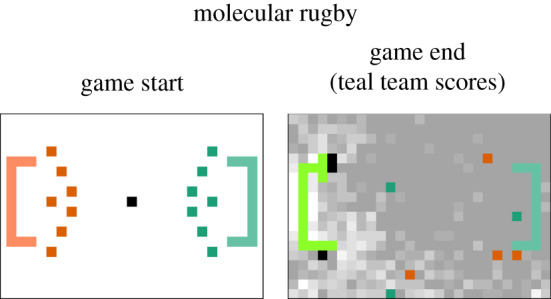
Molecular rugby. Each player submits rules defining how their team interacts with the field. Can you come up with a strategy that beats blind diffusion?

To explore these and other difficulties of molecular design, we propose a new molecular sport—molecular rugby. In molecular rugby, states representing players on two opposing teams, *X* and *Y*, compete to pick up and move a ball to a goal protected by the other team. Fixed transition rules govern basic mechanics (picking up the ball, tackling other players, and scoring). The game is played between teams with various (constrained) transition rules controlling the movement of players on their team. See figure 7 and box 7 for details.

Box 7. **Molecular rugby.**

Molecular rugby is played on a fixed initial field, with a ball, seven *X* players and seven *Y* players, and a goal for each made of *GX* and *GY* states, respectively. The background of the playing field is made up of states *n* ∈ {0, 1, …, 9}, initialized to 0. A set of fixed rules governs the interactions between ball, players, and goals, allowing each team to pick up the ball, tackle other players to steal the ball, and score by bringing the ball to the appropriate goal:

$$\begin{array}{rl} & \;\text{take ball}:\;\left\{\begin{array}{c} X/Y+B\to X/YB+0 \end{array}\right.\\ & \;\text{tackle}:\;\left\{\begin{array}{c} X/YB+Y/X\to X/Y+Y/XB \end{array}\right.\\ & \text{score}:\;\left\{\begin{array}{c} X/YB+GY/X\to WX/Y+GY/X \end{array}\right.\\ & \;\text{win/lose}:\;\left\{\begin{array}{c} GX/Y+WY/X\to WY/X+WY/X\\ X/Y+WX/Y\;\to WX/Y+WX/Y\\ X/Y+WY/X\;\;\to LX/Y+WY/X \end{array}\right. \end{array}$$

Your challenge as a molecular rugby coach is to program interactions between your players (either *X* or *Y*) and the background, i.e. reactions of the form *X* + *n* → *X* + *n*′ or *X* + *n* → *n*′ + *X*, so that your team has a better chance of winning. No other reactions are allowed. The fixed reactions each have rate 10, and you have a total rate budget of 200 to distribute among your team’s reactions.

— We provide two team implementations (a straightforward random-walk implementation and an implementation that changes the field) on our simulator website, in the ‘Molecular Rugby’ example. Can you build a ruleset that beats ours more than half the time?
— In a competition between *K* teams, each team plays each other team *N* times. If one team makes at least $\sqrt{N}$ goals more than the other, it is declared the victor; otherwise the team with the fewer reaction rules is the victor. Host a competition among your friends!
— Invent your own variant of molecular rugby. Perhaps start with different background patterns, such as vertical stripes as yard lines. Perhaps let the background decay like pheromones using reactions 9 → 8, 8 → 7, …. Perhaps have more or less background states. Perhaps allow team players to have more internal states, e.g. *X*1, *X*2, …. Perhaps….

## 7. Conclusion

What, in principle, can chemistry on a surface do?

We have addressed this question with a simple asynchronous cellular-automata-like framework—the surface CRN—that serves as a tractable and comprehensible model of chemistry on a surface. In this model, unimolecular and bimolecular reactions specified in a rule set can occur at any site and between any two neighbouring sites on a surface, respectively. Surface CRNs may not capture the full richness of all possible physics that can occur on a surface surrounded by chemicals, but we claim that they capture many of the important features of chemistry-on-a-surface in much the same way that well-mixed CRNs capture many of the important features of chemistry-in-a-tube.

What can chemistry do on a surface, according to the surface CRN model? The short answer is that chemistry on a surface is Turing-universal, and so in principle can do anything that a computer can do.

More concretely, there are relatively simple and comprehensible surface CRN designs of reaction–diffusion behaviour, direct simulation of synchronous cellular automata, and arbitrary Boolean logic circuits. We have also defined surface CRNs with simple pattern formation and simple swarm behaviours, and we believe that more complex and diverse behaviours are possible with larger rule sets involving more surface reactions.

What constraints, if any, does being on a surface impose on chemistry?

According to the surface CRN model, as we have seen from the manufacturing and swarm examples, computation with just local information and no absolute orientation requires different algorithms compared to that with both local and global information. The differences often lead to either larger numbers of reaction steps to accomplish a task or larger rule sets to compute the information that are not immediately available.

What is surface CRN better at doing than well-mixed CRNs and polymer CRNs?

In well-mixed CRNs, a larger number of specific molecular interactions is required for computing a more complex task. In practice, any compromise in that specificity would result in undesired side reactions that limit the scalability of the CRNs. Surface CRNs trade off complexity of *molecular interaction* for complexity of *spatial placement*—if you can precisely place molecules on a surface, then you can program behaviours with fewer molecular interactions, which will allow skilled chemists to build more complex chemical systems with dynamic and algorithmic behaviours.

As an example, let us compare the logic circuit designs shown in §4 with the seesaw logic circuit scheme described in [10], which is a well-mixed CRN implemented with DNA molecules. The surface CRN implementation requires between two and three times as many reactions for a single logic gate as the seesaw implementation, but, critically, the seesaw gate implementation requires new molecular interactions for every gate in the circuit, while the surface CRN implementation has a fixed set of rules for circuits of any size. The surface CRN’s ability to reuse reactions for multiple logic gates comes directly from its ability to spatially separate components—it replaces chemical specification with spatial separation, which for many systems should prove easier to achieve. Because of the fixed set of rules for arbitrary circuits, undesired side reactions do not scale with the complexity of the circuit. Unlike the well-mixed CRN, in the surface CRN it is not necessary to reduce the side reactions by reducing concentrations of chemical species, allowing each reaction to be as fast as possible regardless of the circuit size.

Moreover, surface CRNs can be massively parallel. Billions to trillions of tiny surfaces could float around in one test tube, each carrying out a distinct computation. By contrast, a well-mixed CRN in one test tube can compute exactly one thing at any time. In principle, polymer CRNs can be parallel [37], but existing constructions of polymer CRNs using DNA nanotechnology require a single copy of certain polymers in order to be Turing-universal [27–29], severely limiting their capability for parallel computation.

The theoretical understanding that we have explored in this work will hopefully inspire construction of increasingly interesting chemical systems on surfaces. There already exist systematic methods for implementing arbitrary well-mixed CRNs using DNA nanotechnology, both in theory [91] and practice [13,14], as well a theoretical approach for implementing arbitrary surface CRNs using DNA strand displacement reactions attached to a DNA origami surface [38]. Thus, the surface CRN is more than a theoretical tool for understanding the abstract properties of surface chemistry—it can, in principle, be physically implemented in a real-world laboratory. These implementations will provide a starting point for the development of complex, programmed molecular systems that may someday be extended to other types of molecules including RNA and proteins.

Beyond guiding efforts to engineer molecular machines, studying the surface CRN model will also help answer a fundamental question: what capabilities and constraints of molecular interactions guided the origin and evolution of life? While surface CRNs are more scalable and parallel than well-mixed and polymer CRNs, no single type of chemistry alone provides the sole solution for life-level complexity. The receptors on cell membranes represent an example of surface chemistry. The filaments in cytoskeletons represent an example of polymer chemistry. The transcription factors in genetic regulatory circuits represent an example of well-mixed chemistry. Understanding the advantages and trade-offs of each type of chemistry will clarify the design principles for both engineered and natural molecular systems, for example defining what the best geometry (or combination of geometries) is for solving a given molecular task. Naturally, this will require investigation of more complex possibilities—including three-dimensional geometry, reconfiguration between different geometries, and integration of different geometries—and will put us on the road toward understanding and engineering molecular systems with behaviours as sophisticated as those seen in biology.

## Data accessibility

Simulator code, including examples used in this paper, can be found in the ‘paper-release’ branch of the GitHub repository at https://github.com/sclamons/surface_crns, a fork of which is also available from the DNA and Natural Algorithms Group on GitHub (https://github.com/DNA-and-Natural-Algorithms-Group). A browser interface to the simulator is also available, along with examples from this paper and others, at http://www.dna.caltech.edu/Surface_CRN_Simulator/.
